# The Crosstalk between Nrf2 and Inflammasomes

**DOI:** 10.3390/ijms19020562

**Published:** 2018-02-13

**Authors:** Paulina Hennig, Martha Garstkiewicz, Serena Grossi, Michela Di Filippo, Lars E. French, Hans-Dietmar Beer

**Affiliations:** 1 Department of Dermatology, University Hospital of Zurich, Gloriastrasse 31, F30, CH-8091 Zurich, Switzerland; Paulina.Hennig@usz.ch (P.H.); Martha.Garstkiewicz@usz.ch (M.G.); Serena.Grossi@usz.ch (S.G.); Michela.Difilippo@usz.ch (M.D.F.); Lars.French@usz.ch (L.E.F.); 2 Faculty of Medicine, University of Zurich, CH-8091 Zurich, Switzerland

**Keywords:** caspase-1, Nrf2, inflammasome, nucleotide-binding oligomerization domain (NOD)-like receptor containing pyrin domain 3 (NLRP3), inflammation

## Abstract

The Nrf2 (nuclear factor E2-related factor or nuclear factor (erythroid-derived 2)-like 2) transcription factor is a key player in cytoprotection and activated in stress conditions caused by reactive oxygen species (ROS) or electrophiles. Inflammasomes represent central regulators of inflammation. Upon detection of various stress factors, assembly of the inflamasome protein complex results in activation and secretion of proinflammatory cytokines. In addition, inflammasome activation causes pyroptosis, a lytic form of cell death, which supports inflammation. There is growing evidence of a crosstalk between the Nrf2 and inflammasome pathways at different levels. For example, Nrf2 activating compounds inhibit inflammasomes and consequently inflammation. This review summarizes what is known about the complex and predominantly antagonistic relationship of both stress-activated pathways.

## 1. Introduction

Many cells of our body are regularly exposed to various kinds of stress. This is particularly—but not only—true for epithelial cells, such as those of the skin, which are in permanent contact with the environment [1]. Upon detection of stress factors, cells activate different pathways, which help them and the affected tissue to cope with the stressor and to restore a new homeostatic state. This can result in the induction of inflammation, which is considered to be an attempt of the tissue to restore a stress-free state [2]. Both the Nrf2 (nuclear factor E2-related factor or nuclear factor (erythroid-derived 2)-like 2) system and inflammasomes represent stress-activated pathways [3,4]. Nrf2 is a transcription factor, whose activation results in expression of cytoprotective proteins and enzymes, which support the survival of Nrf2-activating cells under stress conditions, for example during inflammation [5,6]. In contrast, inflammasome activation induces inflammation, which might be beneficial for the tissue (this is true for acute but not for chronic inflammation) and the whole organism. However, inflammasome activation causes death of the cells, in which the inflammasome has been activated. Therefore, both stress-induced and inflammation-associated pathways are beneficial for the tissue. However, the consequences of their activation at the level of the individual cells are opposite. This review addresses the complex relationship of the Nrf2 and inflammasome pathways. Both are associated with many inflammatory conditions, including acute and chronic inflammation. In addition, they are linked to reactive oxygen species (ROS) and autophagy. Finally, the Nrf2 system regulates expression of downstream genes of inflammasomes, and both complexes might physically interact. The fact that Nrf2 and inflammasomes are connected to each other at different levels and that they are involved in many and common inflammatory diseases points to a special importance of the crosstalk of both pathways.

After an introduction of the Nrf2/Keap1 system (Section 2, Section 3 and Section 4) and inflammasomes (Section 5 and Section 6), a description of the crosstalk of both pathways follows (Section 7 and Section 8). Possible molecular mechanisms underlying or contributing to this crosstalk are also discussed (Section 9).

## 2. The Nrf2 Transcription Factor

**Figure 1:**
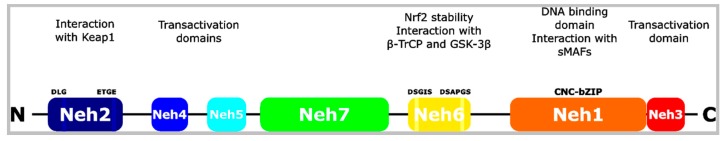
Structure of the Nrf2 (nuclear factor E2-related factor or nuclear factor (erythroid-derived 2)-like 2) transcription factor. Nrf2 consists of seven Neh domains. Neh1 is the CNC-bZIP domain mediating DANN binding and interaction with sMAFs. The latter are required for transcription. Neh3, 4 and 5 are transactivation domains. Neh6 is rich in serine residues and regulates the stability of Nrf2. β-TrCP interacts with Nrf2 via this domain, particularly after phosphorylation by GSK-3β. Neh2 mediates the interaction and regulation with Keap1 via the DLG and ETGE motifs. This binding results in ubiquitination of Nrf2 and in its proteasomal degradation. bZIP: basic leucine zipper. CNC: Cap’n’collar. GSK-3β: glycogen synthase kinase-3β. Keap1: Kelch-like ECH-associated protein 1. Neh: Nrf2-ECH homology. sMAF: small musculoaponeurotic fibrosarcoma. β-TrCP: β-transducing repeat-containing protein. Adopted and modified from [6].

Nrf2 is a key regulator of expression of cytoprotective genes required for stress resistance [3,6,7]. It belongs to the Cap’n’collar family of bZIP (basic leucine zipper) transcription factors. Transcription of the Nrf2 coding *Nfe2l2* gene is induced by a positive feedback loop mediated by Nrf2, by NF-κB. by the aryl hydrocarbon receptor upon binding to polycyclic aromatic hydrocarbons, as well as by several oncogenic pathways [6]. Although *Nfe2l2* spans only 2.2 kb, the protein migrates at an apparent size of more than 100 kDa by SDS-PAGE for unknown reasons [8]. Nrf2 consists of seven Neh (Nrf2-EHC homology) domains, which regulate its activity by binding to other proteins or to DNA (Figure 1). The Neh1 domain is essential for Nrf2’s transcriptional activity, since it contains the bZIP DNA binding region and mediates interaction with sMAF (small masculoaponeurotic fibrosarcoma) proteins [9]. Upon sMAF binding, Nrf2 targets so-called AREs (antioxidant response elements) in the promoter region of several hundred genes, including many that code for cytoprotective proteins [7]. These gene products include essential proteins of the glutathione (e.g., glutamate-cysteine ligase) and thioredoxin (e.g., thioredoxin reductase) system, which comprise the most important cellular redox buffers [10]. In addition, Nrf2 regulates genes whose products are required for detoxification of ROS and xenobiotics (e.g., NQO1 (NAD(P)H dehydrogenase [quinone] 1)), NADPH regeneration (e.g., glucose-6-phosphate dehydrogenase), and heme and iron metabolism (e.g., HO-1 (heme oxygenase 1)) [6]. Since inflammation is associated with oxidative stress, the Nrf2 pathway is believed to play an important role in the pathogenesis of cancer and common inflammatory and neurodegenerative diseases [11,12]. In general, Nrf2 protects from infection, and an inverse correlation between infection and a decline in Nrf2 activity has been demonstrated [13]. For example, viruses, such as hepatitis C virus or HIV, inhibit or decrease Nrf2 [14,15]. In contrast, Marburg virus and hepatitis B virus induce Nrf2 expression [16,17,18]. Recently, it has been established that Nrf2 directly prevents transcription of genes encoding the pro-inflammatory cytokines IL-6, proIL-1β and proIL-1α (see also Section 9.3), although the underlying molecular mechanisms are incompletely understood [7,19].

In addition, to the above-mentioned modes of regulation of Nrf2 and—in turn of Nrf2 target gene expression, other mechanisms of control of Nrf2 activity are important. Most of these mechanisms regulate Nrf2 activity upon its protein stability, which is reflected by Nrf2’s short half-life of only 10–30 min under homeostatic conditions [6]. 

## 3. Canonical Nrf2 Activation

Keap1 (Kelch-like ECH-associated protein 1) is the most important regulator of Nrf2 activity [3,7]. In the cytoplasm, two molecules of this E3 ubiquitin ligase substrate adaptor bind to the amino terminal Neh2 domain of Nrf2 and mediate its polyubiquitination by interaction with the E3 ubiquitin ligase complex Cul3/Rbx1 (Cullin 3/RING-box protein 1). This results in constant Nrf2 degradation by the proteasomal pathway. Small amounts of Nrf2 escape Keap1-dependent degradation, causing constitutive and weak expression of Nrf2 target genes upon nuclear translocation. The Keap1-dependent ubiquitin ligase activity is regulated in a redox-sensitive manner. Oxidative stress or electrophiles oxidize specific cysteine residues of Keap1, causing a conformational change of the adaptor protein and an inhibition of the E3 ubiquitin ligase activity, although Cul3 remains bound to Keap1 [7]. Then, newly synthesized Nrf2 bypasses Keap1, translocates to the nucleus and induces target gene expression. Nrf2 activation upon oxidation of cysteine residues of Keap1 is termed canonical Nrf2 activation [20]. Interestingly, the oxidation of Keap1 by the many different Nrf2 activators seems to be a highly specific process. These activators can be grouped into different classes, depending on the preferentially targeted cysteine residues of Keap1. For example, the Nrf2 activator sulforaphane (SFN), which is a component of broccoli sprouts, or dimethyl fumarate (DMF), an approved anti-inflammatory drug for the treatment of patients suffering from psoriasis or multiple sclerosis, oxidize mainly cysteine 151 of Keap1 [21,22].

## 4. Non-Canonical Nrf2 Activation

Binding of Keap1 to p62/SQSTM1 (sequestosome 1) causes non-canonical Nrf2 activation [20,23]. p62 is a multi-domain and multi-functional protein that protects cells from stress by autophagic clearance and Nrf2 activation [24]. It acts as a cargo receptor for the autophagic machinery and its degradation correlates with autophagic flux [25]. The Kir domain of p62 binds to the same stretch of amino acids of Keap1 as Nrf2 and, thereby, releases Nrf2 from its inhibitor, resulting in expression of Nrf2 target genes. In autophagy-deficient cells, phosphorylated p62 aggregates with Keap1 in the cytoplasm, causing constant Nrf2 activation [26,27,28]. This results in a positive feedback loop as Nrf2 induces p62 expression [25]. In addition, p62 regulates NF-κB that, in turn, increases Nrf2 expression.

In different types of cancer, Nrf2 is activated as a consequence of epigenetic silencing of the *Keap1* gene by promoter methylation [6,29,30]. Increased Nrf2 target gene expression supports the stress resistance of the cancer cells and induces changes in metabolic pathways [31].

PGAM5 (phosphoglycerate mutase family member 5) is a mitochondrial phosphatase that was originally shown to interact with Keap1 resulting in its Keap1-dependent ubiquitination and subsequent proteasomal degradation [32]. Later, it turned out that PGAM5 also interacts with Nrf2 and negatively regulates Nrf2’s transcriptional activity [33]. Interestingly, PGAM5 is a central player in necroptotic cell death [34]. In addition, several other proteins, such as DPP3 (dipeptidyl-peptidase 3) or WTX (Wilms tumor gene on X chromosome), can interact with Keap1 and modulate the Nrf2-Keap1 pathway [6].

Nrf2 activation upon perturbation of the Nrf2-Keap1 complex can also be mediated by Nrf2 targeting. PKC (protein kinase C) phosphorylates Nrf2 at serine 40, causing its dissociation from Keap1, translocation to the nucleus, and induction of target gene expression [35,36]. This has been shown to occur in response to oxidative stress, which activates PKC and most likely supports canonical Nrf2 activation [37].

Another example is p21, a major p53 target and cell cycle inhibitor, which interacts with Nrf2 and thereby liberates it from Keap1 [38]. This p21-mediated Nrf2 activation antagonizes anti-cancer drugs in a murine model of skin cancer [39].

## 5. Inflammasomes

**Figure 2:**
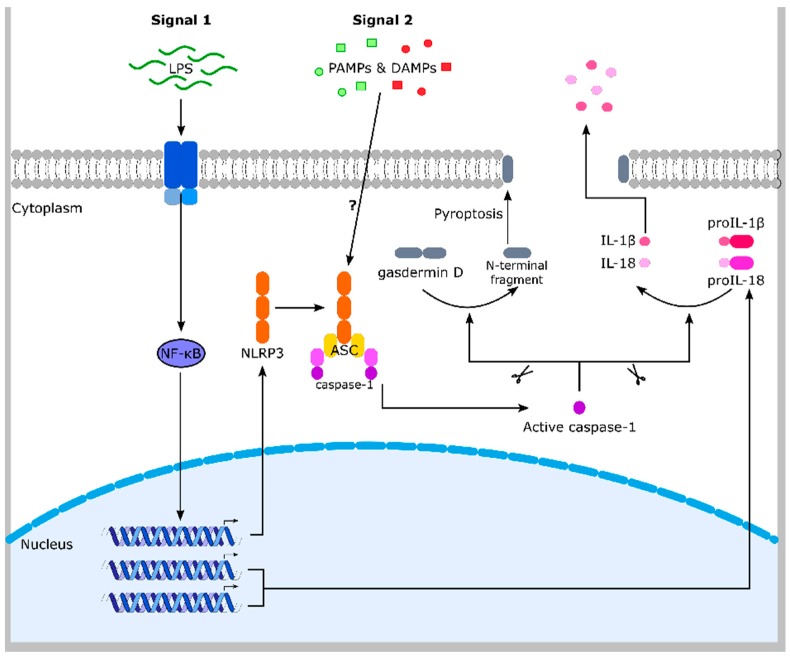
The NLRP3 inflammasome. LPS (lipopolysaccharide) (and other compounds) induces TLR4 (Toll-like receptor 4)-dependent activation of the transcription factor NF-κB, resulting in NLRP3, proIL-1β and proIL-18 expression. By an unknown mechanism, NLRP3 senses different PAMPs and DAMPs, which induce NLRP3 inflammasome assembly (NLRP3, ASC and caspase-1). Caspase-1 is activated and cleaves the pro-inflammatory cytokines proIL-1β and -18. Secretion of the mature and active cytokines induces inflammation. Caspase-1-dependent cleavage of gasdermin D causes pore formation in the plasma membrane upon oligomerization of its N-terminal part. This facilitates IL-1β and -18 release and causes a lytic form of cell death (pyroptosis), which supports inflammation. The NLRP3 is one of several similar inflammasome complexes. ASC: apoptosis-associated speck-like protein containing a CARD (caspase activation and recruitment domain). DAMPs: danger-associated molecular patterns. IL: interleukin. NLRP3: Nod-like receptor family pyrin domain containing 3. NF-κB: nuclear factor κ-light-chain-enhancer of activated B-cells. PAMPs: pathogen-associated molecular patterns.

Caspases are cysteine proteases that play essential roles in apoptosis [40,41]. They act either upstream as initiators of apoptosis—in this case they possess a long prodomain—or downstream as executioners. Initiator caspases are activated in large molecular complexes due to protein-protein interactions mediated by their prodomain. Caspase-1 does also contain a large prodomain (a CARD (caspase recruitment domain)) but is rarely associated with apoptosis. In contrast, caspase-1 plays a fundamental role in inflammation [4]. It is the central molecule of multimeric protein complexes, termed inflammasomes. These complexes include an upstream sensor protein such as NLRP3 (nucleotide-binding oligomerization domain (NOD)-like receptor containing pyrin domain 3), the adaptor protein ASC (apoptosis-associated speck-like protein containing a CARD), and caspase-1 (Figure 2). Whereas NLRP3, NLRP1, AIM2 (absent in melanoma 2), NLRC4 (NLR family CARD domain containing protein 4), and pyrin are well established inflammasome sensors, more sensors have been identified during the last years [42]. These sensors identify certain stress factors, such as pathogens, but also endogenous molecules, and subsequently induce the oligomerization of ASC in large complexes, called ASC specks. Upon caspase-1 binding, the protease is activated by an autocatalytic process and in turn activates the proinflammatory cytokines proIL-1β and -18. Secretion of the mature and active forms of the cytokines induces inflammation [4]. In addition, caspase-1 cleaves gasdermin D, whose N-terminal fragment translocates to the outer membrane where it forms large pores resulting in a lytic form of cell death (pyroptosis) [43,44]. Inflammasomes are associated with numerous autoimmune, autoinflammatory, metabolic, and infectious diseases [4,42].

## 6. The NLRP3 Inflammasome and ROS

Even before the identification of inflammasomes [45] it was known that mutations of the NLRP3 encoding gene cause an autoinflammatory disease characterized by fever and urticaria-like skin lesions [46]. Meanwhile an involvement of the NLRP3 inflammasome in many inflammatory diseases is discussed [4,47]. In addition, the NLRP3 inflammasome is activated by many different stress factors as well as upon non-canonical inflammasome activation mediated by gasdermin D [44]. However, it is still a matter of debate which molecular mechanisms govern NLRP3 inflammasome activation [42]. It is accepted that two signals are involved (Figure 2). LPS (lipopolysaccharide) stimulation of immune cells represents a well established “signal one” that induces expression of NLRP3, proIL-1β and proIL-18, mediated by NF-κB activation downstream of TLRs [42]. Then, danger- and pathogen-associated molecular patterns (DAMPs, PAMPs) can activate the NLRP3 inflammasome (“signal two”), including ATP, released from injured and dying cells. Interestingly, regulatory T cells (T_regs_) express only low levels of Nrf2 and undergo apoptosis induced by high levels of ROS in the tumor environment [48]. These dying T_regs_ convert ATP to adenosine, which strongly enhances their ability to suppress T cell activation and avoids inflammasome activation [48]. The discussed mechanism(s) underlying “signal two” are diverse and include changes in ion homeostasis, lysosomal rupture, cardiolipin or a role of mitochondrial DNA [42,49]. In addition, a role of ROS in NLRP3 inflammasome activation has been suggested.

Many different and structurally diverse molecules activate the NLRP3 inflammasome, and it is very unlikely that this is mediated in all cases by a direct physical interaction with NLRP3. Therefore, it was hypothesized that ROS, whose generation often occurs in cells exposed to these molecules, integrate all these signals and induce NLRP3 inflammasome activation [50,51]. How NLRP3 senses changes in ROS is not known, but two different pathways have been suggested [52]. TXNIP (thioredoxin-interacting protein), which is bound to thioredoxin under homeostatic conditions, is liberated by ROS and can then interact with NLRP3 resulting in inflammasome assembly and activation [53]. There are different sources of ROS. The most important are NADPH oxidases and mitochondria, and a role of the latter in inflammasome activation has been suggested [54]. The mitochondrial adaptor protein MAVS (mitochondrial antiviral signaling protein) associates with NLRP3, localizes it to mitochondria, and supports NLRP3 inflammasome activation [55]. There are also very interesting reports suggesting a biphasic and more complex role of the cellular redox state in inflammasome activation [56]. In addition, the NLRP3 inflammasome is blocked in mice lacking expression of SOD1 (superoxide dismutase 1), which are characterized by strongly increased ROS levels [57]. However, it should be pointed out that a contribution of ROS to NLRP3 inflammasome activation is controversially discussed and might be species-, cell type-, context-, and stimulus-dependent [52].

## 7. Nrf2 Expression Supports Inflammasome Activation

Atherosclerosis is associated with ROS causing lipid oxidation and its accumulation in the arterial wall. This induces recruitment of leukocytes and inflammation, where particularly IL-1 is supposed to play a central role [58,59]. Consequently, it can be anticipated that Nrf2 and its activation, which induces expression of ROS detoxifying proteins, ameliorates atherosclerosis. Surprisingly, the opposite is true, as ablation of Nrf2 expression protects from atherosclerosis in a mouse model of this disease [58,60]. Cholesterol crystals, which are found in atherosclerotic lesions, are able to activate the NLRP3 inflammasome, which is required for atherogenesis [58,61]. Similarly, an inflammasome-dependent model of chronic kidney disease is also dependent on Nrf2 expression [62]. Later it was shown that Nrf2 expression supports NLRP3 and AIM2 inflammasome activation in murine immune cells, NLRP1/NLRP3 inflammasome activation in human primary keratinocytes and the latter inflammasome type also in THP-1 cells [63,64,65]. In contrast, NLRC4 inflammasome activation is not affected by Nrf2 ablation [64]. Mechanistically, a physical crosstalk between both pathways has been suggested [63], and Nrf2 has been shown to colocalize with ASC specks [64]. Although Nrf2 ablation only dampens inflammasome activation rather than completely inhibiting it, these results demonstrate an unexpected proinflammatory role of the transcription factor [63,66,67]. Interestingly, Nrf2 is quickly degraded upon inflammasome activation [63]. 

## 8. Nrf2 Activation and Inflammasome Inhibition 

**Figure 3:**
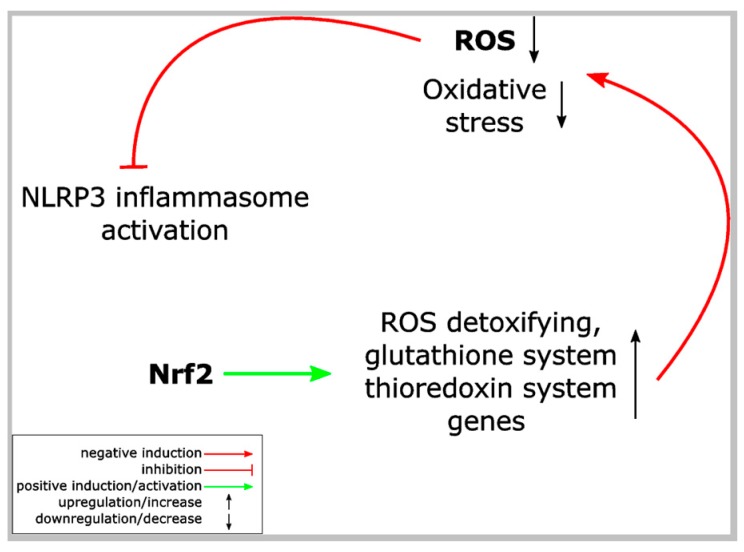
ROS links Nrf2 and NLRP3 inflammasome activation. Proposed model of NLRP3 inflammasome inhibition by Nrf2 activation. Nrf2 activation, e.g., upon modification of regulatory cysteine residues of its inhibitor Keap1, results in expression of several different proteins, which detoxify ROS. Since ROS is required for NLRP3 inflammasome activation, this detoxification antagonizes the NLRP3 inflammasome and inflammation. ROS: reactive oxygen species.

In general, Nrf2 activation is considered to have anti-inflammatory effects, although Nrf2 target genes are not directly—for example via regulation of genes encoding proinflammatory cytokines—involved in inflammation [3,7]. However, inflammation is associated with oxidative stress and ROS, which is required for the destruction of or prevention from invasion of pathogens. It is reasonable to assume that Nrf2 activation limits the negative effects of ROS for the cells of an inflamed tissue and, eventually, inflammation. In addition, the assumption that NLRP3 inflammasome activation is regulated by ROS and oxidative stress offers an even more direct explanation for the anti-inflammatory activity of Nrf2 signaling (Figure 3) [50]. Consequently, since the presentation of the concept that ROS regulates NLRP3 inflammasome activation, several studies have been published, which demonstrate a correlation between Nrf2 activation and NLRP3 inflammasome inhibition in many different disease models associated with inflammation (Table 1). Most of these studies used substances isolated from plants, known in the Eastern, traditional Chinese medicine for their efficacy for patients suffering from inflammatory conditions. It is not known whether all these compounds are canonical Nrf2 activators, but for some of them this is well established (Table 1). Nevertheless, in each case treatment of cells or animals with these compounds causes Nrf2 activation and NLRP3 inflammasome inhibition. Since most of the studies are based on animal models for inflammatory diseases, where whole tissues were analyzed, in most cases it is not identified whether Nrf2 activation and NLRP3 inflammasome repression occur in the same cells, in neighboring cells, or even in different cell types. In addition, about one third of the studies demonstrate a reduction of NF-κB activation in association with Nrf2 activation and NLRP3 inflammasome inhibition. However, from these publications (Table 1) it cannot be concluded that NLRP3 inflammasome inhibition is beneficial in the corresponding diseases in human. This would be the case, if indeed the pathological situation in human would rely on the NLRP3 inflammasome rather than only the (artificial) induction of the disease state in the animal model. In conclusion, the high number of reports demonstrating anti-inflammatory effects of Nrf2-activating substances in different disease models associated with NLRP3 inflammasome inhibition (and in part with NF-κB inhibition) suggests that the crosstalk between these pathways is of high relevance. 

## 9. Possible Mechanisms Underlying the Crosstalk between Nrf2 and Inflammasomes

### 9.1. Nrf2 Activation and Inflammasome Inhibition

Why is Nrf2 activation accompanied with NLRP3 inflammasome inhibition in so many different inflammatory disease models (Table 1)? Substances such as sulforaphane, prostaglandin 15d-PGJ_2_ or curcumin are well-established Nrf2 activators via inhibition of its regulator Keap1 upon covalent modification of regulatory cysteine residues (canonical Nrf2 activation) [3,5]. In contrast, xanthohumol for example is supposed to stabilize Nrf2 independently of Keap1, but activates AMPK (p38 MAP kinase), which in turn inhibits Gsk-3β (glycogen synthase kinase 3β) [68]. This enhances Nrf2 activity, since phosphorylation of the transcription factor in the nucleus by Gsk-3β causes its Keap1-independent degradation [69]. Arsenic activates Nrf2 upon the inhibition of autophagy and the accumulation of p62. Then, p62 binds to Keap1 and liberates Nrf2 [20]. The molecular mechanisms underlying Nrf2 activation caused by several other substances is only poorly defined (Table 1). Most authors argue that Nrf2 activation—irrespective of the underlying mechanisms—induces upregulation of antioxidant target genes, whose expression causes a reduction of intracellular ROS levels (Figure 3). In turn, this inhibits NLRP3 inflammasome activation, which is caused by ROS-induced dissociation of thioredoxin from TXNIP and the binding of the latter to NLRP3 supporting inflammasome activation [53,54]. If this order of events underlies NLRP3 inflammasome inhibition by Nrf2 activators, then inflammasome inhibition should be dependent on Nrf2 expression, the induction of Nrf2 target gene expression, and a reduction in ROS. In general, it is well known that Nrf2 knockout mice suffer from a more severe phenotype in different inflammatory disease models compared to wild type mice [70]. Concerning NLRP3 inflammasomes, a knockdown of Nrf2 expression enhanced NLRP3 inflammasome activity in a model of cerebral ischemia reperfusion injury and in brain injury after intracerebral hemorrhage [71,72]. In addition, it was reported that the anti-inflammatory effect of sulforaphane is reduced in THP-1 cells upon a knockdown of Nrf2 expression [73] and that the protective effect of Nrf2 activation in a colitis model is dependent on NLRP3 expression [74].

Interestingly, there is also evidence for anti-inflammatory activity of Nrf2-activating compounds independently of Nrf2 expression, Nrf2 target gene expression, and Nrf2-dependent ROS regulation. The Nrf2 activating compounds sulforaphane and 15d-PGJ_2_ also inhibit NLRP3 inflammasome activation in Nrf2 knockout cells [63,66,67]. Additionally, sulforaphane inhibits NLRP3 inflammasome activation even in Keap1 knockout cells [63]. DMF, which is used as a drug for patients suffering from multiple sclerosis, has anti-inflammatory activity in a mouse model of experimental autoimmune encephalomyelitis, irrespectively of Nrf2 expression [75]. The Nrf2 activator xanthohumol is protective in a mouse model of acute lung injury, and this effect is only slightly reduced in Nrf2 knockout mice [68]. An involvement of ROS is only discussed for NLRP3 inflammasome activation, but sulforaphane blocks also AIM2, NLRP1, and NLRC4 inflammasome activation [67]. In addition, neither expression of Nrf2 target genes nor protein synthesis is needed for inflammasome inhibition by sulforaphane [63,67].

These results suggest that Nrf2 activators do not only target Nrf2 via covalent modifications of Keap1’s cysteine residues, but also other pathways. An obvious candidate is NF-κB (Table 1), which is linked to Nrf2 in a complex manner [6,70]. NF-κB activation is required for NLRP3 inflammasome priming and particularly for induction of proIL-1β expression [42]. Interestingly, several publications suggest a suppression of proIL-1β expression and priming by Nrf2 activating compounds [74,76,77,78,79,80]. In addition, it is known that strong Nrf2 activating electrophiles, such as 15d-PGJ_2_, can also inhibit the NF-κB pathway [70,81,82].

It is also conceivable that Nrf2 activating compounds directly target inflammasome proteins. It has been excluded that caspase-1 is modified, as the purified enzyme cannot be inhibited by sulforaphane [67]. Since sulforaphane prevents ASC speck formation, it is more likely that inflammasome activation is inhibited upstream of inflammasome assembly [63]. During the last years, several factors, which contribute to inflammasome activation and which represent principal targets for inflammasome inhibition by Nrf2 activating compounds, have been identified [42,83].

### 9.2. Non-Canonical Nrf2 Activation and Inflammasome Inhibition

**Figure 4:**
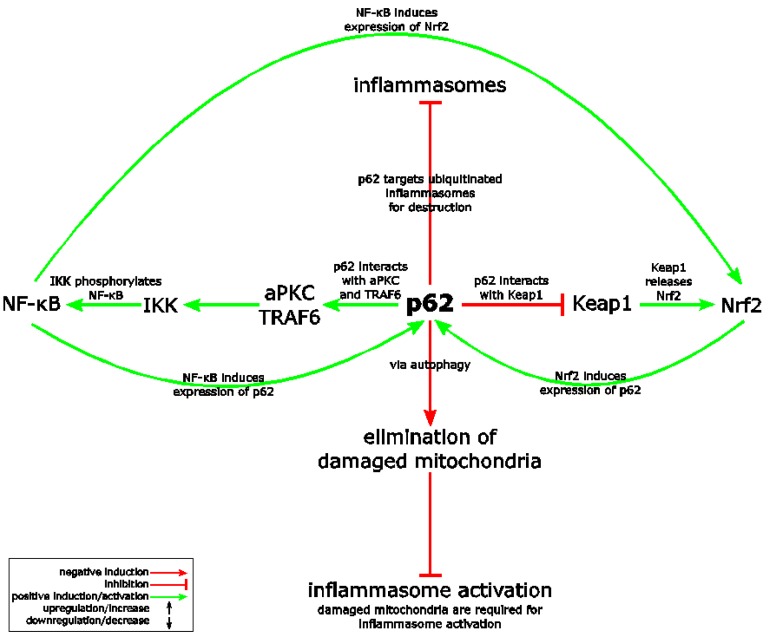
SQSTM1/p62 (sequestosome-1) connects Nrf2, the NLRP3 inflammasome and NF-κB. p62 causes non-canonical Nrf2 activation via interaction and destruction of Keap1. In addition, p62 is a well-known activator of NF-κB. NF-κB as well as Nrf2 induce p62 and Nrf2 expression (positive feedback loop). In contrast, p62 antagonizes the NLRP3 inflammasome pathway by its targeting of ubiquitinated inflammasome components to autophagosomes. In addition, p62 supports the elimination of damaged mitochondria, which are believed to support NLRP3 inflammasome activation. See Section 9.2 for details. aPKC: atypical protein kinase C. IKK: IκB kinase. TRAF6: tumor necrosis factor receptor-associated factor 6.

The multi-functional and multi-domain protein p62 is supposed to be a critical regulator of the Nrf2, NF-κB and NLRP3 inflammasome pathways (Figure 4) [25,84,85]. Since p62 supports Nrf2 and NF-κB activity but antagonizes the NLRP3 inflammasome, p62 might represent a link between the three pathways responsible for their crosstalk. Originally, p62 was identified as an important mediator of NF-κB [25]. With its aminoterminal PB1 domain, which is also required for self-oligomerization, p62 interacts with aPKC (atypical protein kinase C). In addition, p62 binds TRAF6 via its TRAF6 binding domain and it can also activate RIP1 [25]. In turn, IKK (Iκ B kinase) is activated, which induces NF-κB translocation upon phosphorylation and inhibition of its inhibitor IκBα (Figure 4). However, knockdown or ablation of p62 also antagonizes Nrf2 (see above in Section 4) and supports inflammasome activation [84,85]. Inflammasome activation in macrophages triggers autophagosome formation [84]. Inflammasome activation and IL-1β production is limited upon Lys63-linked polyubiquitination of inflammasomes, followed by autophagosomal delivery upon interaction with the autophagic adaptor p62 [84]. Alternatively or in addition, it has been reported that p62 is required for the elimination of damaged mitochondria via autophagy (Figure 4) [85]. It is believed that NLRP3 inflammasome agonists induce inflammasome complex assembly upon damage of mitochondria and the release of inflammasome activating signals [85]. Upon p62-dependent destruction of these damaged mitochondria, NLRP3 inflammasome activation is restricted. Oxidation of cysteine residues of p62 by a poorly characterized mechanism increases autophagy [86]. However, it is not known, whether p62 is also regulated by oxidation with Nrf2 activating compounds [86]. Interestingly, NF-κB has a central role in controlling this crosstalk. Its activation is not only required for priming of the NLRP3 inflammasome (induction of NLRP3 and proIL-1β expression), but also for the induction of p62 expression, which limits inflammasome activation. As p62 supports NF-κB as well as Nrf2 activation, a positive feedback loop results via induction of p62 expression by Nrf2 (Figure 4). It is likely that this loop is more relevant before than after inflammasome assembly, since Nrf2 is quickly degraded upon inflammasome activation [63].

**Figure 5:**
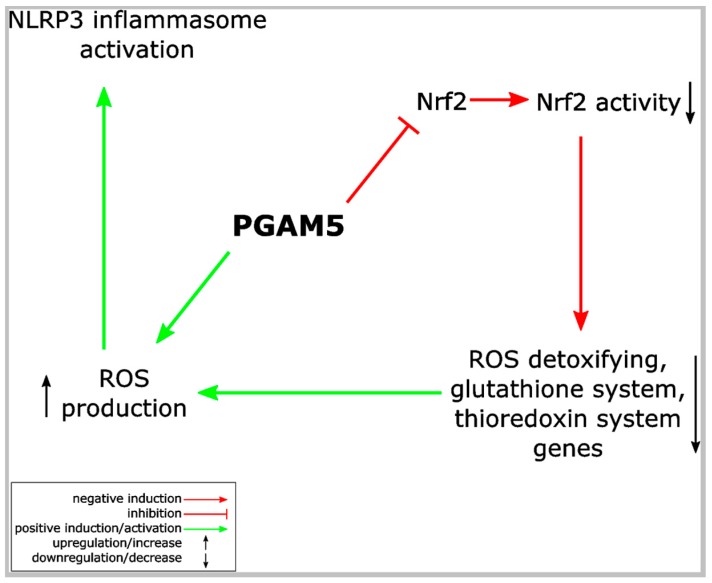
PGAM5 links the NLRP3 inflammasome and Nrf2 pathway. PGAM5 inhibits Nrf2’s transcriptional activity and, thereby, suppresses expression of ROS detoxifying proteins. This increase in ROS might support NLRP3 inflammasome activation. PGAM5 expression is required for NLRP3 inflammasome activation. See Section 4 and Section 9.2 for details. PGAM5: serine/threonine-protein phosphatase PGAM5, mitochondrial, phosphoglycerate mutase family member 5.

The phosphatase PGAM5, which is able to interact with Keap1 as well as with Nrf2, is an inhibitor of Nrf2’s transcriptional activity (see above in Section 4). In contrast, it supports necroptotic cell death [34], a form of cell death termed oxeiptosis [87], and promotes inflammasome activation in macrophages, which causes pyroptosis [88]. Therefore, PGAM5 could play a role in the antagonistic crosstalk between Nrf2 and the NLRP3 inflammasome (Figure 5).

### 9.3. Transcriptional Repression by Nrf2

As mentioned above, the general opinion is that Nrf2 dampens inflammation as an indirect consequence of its role in the regulation of antioxidative and ROS-detoxifying genes, such as NQO1, HO-1 or enzymes and proteins associated with the glutathione and thioredoxin system (see in Section 2 and Figure 3). Interestingly, it has been recently suggested that Nrf2 is directly involved in the regulation of expression of proinflammatory genes [19]. These genes include those encoding the inflammasome effector proteins proIL-1β and proIL-1α—the latter is among others also secreted upon inflammasome activation—as well as IL-6, which is often released downstream of IL-1 [19,89]. The molecular mechanisms underlying this novel activity of Nrf2 are only partially characterized [7]. However, Nrf2 activation in macrophages upon a knockout of Keap1 expression or treatment of cells with strong electrophiles, such as diethyl maleate or 15d-PGJ_2_, suppressed the expression of a number of genes, including *IL6*, *IL1b*, *IL1a* and *IL12b* [19]. Chromatin immunoprecipitation (ChIP)-seq and ChIP-qPCR revealed that Nrf2 binds in regions proximal to the promoter of these genes and inhibits their LPS-induced expression. This binding of Nrf2 occurs independently of ROS and does not require the known Nrf2-binding motifs [19]. It has been suggested that Nrf2 binding inhibits recruitment of RNA Polymerase II to the transcriptional start site of the above-mentioned pro-inflammatory genes and thereby suppresses their expression, since binding of other transcription factors was not affected. A reduction of proIL-1β protein expression upon a knockout of Keap1 expression in THP-1 cells was also reported in a second publication, as was suppression of NLRP3 expression [63]. Although these data suggest for the first time a direct anti-inflammatory activity of Nrf2, they have to be carefully interpreted before the underlying molecular mechanisms are elucidated. It is important to keep in mind that Nrf2 activating compounds might have several additional independent effects of Keap1 and Nrf2 targeting other proteins and pathways (see in Section 9.1). In addition, Keap1 is not only a regulator of Nrf2, but interacts with several other proteins. Nevertheless, transcriptional repression of proinflammatory and NLRP3 inflammasome-associated genes by Nrf2 or, in other words, NLRP3 inflammasome priming, represents a very attractive explanation for the anti-inflammatory activity of this transcription factor.

## 10. Conclusions

**Figure 6:**
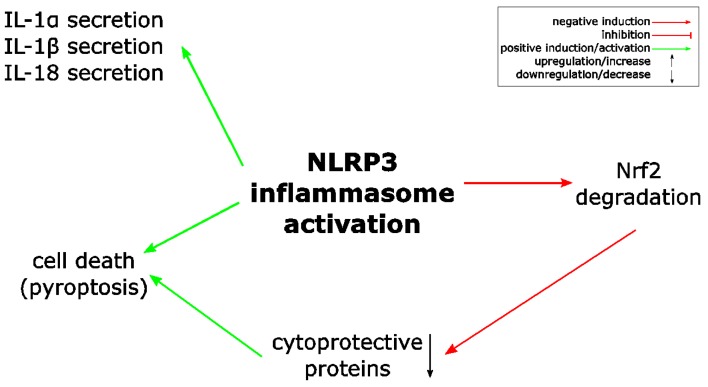
NLRP3 (Nucleotide-binding oligomerization domain (NOD)-like receptor containing pyrin domain 3) inflammasome activation causes Nrf2 degradation. Activation of the NLRP3 inflammasome causes rapid Nrf2 protein degradation by an unknown mechanism [63].

The Nrf2 transcription factor and the NLRP3 inflammasome are both associated with stress and inflammatory conditions. Whereas NLRP3 inflammasome activation induces inflammation and eventually death of the inflammasome-activating cell, Nrf2 activation supports cell survival and rather prevents inflammation. During the last few years, a crosstalk and inverse correlation of both pathways in regulating inflammation became evident. However, the underlying molecular mechanisms are only partially understood. ROS represents a link between both pathways, since proteins expressed upon Nrf2 activation detoxify ROS, whereas ROS is supposed to activate the NLRP3 inflammasome [3,50]. Therefore, it is reasonable to assume that Nrf2 activation causes NLRP3 inflammasome inhibition. The transcription factor NF-κB is also associated with both pathways. NF-κB is required for priming of the NLRP3 inflammasome [42] and it also induces Nrf2 expression [6]. Most importantly, some Nrf2 activating compounds suppress NF-κB activation independently of the Nrf2/Keap1 pathway [70,81,82]. In addition, Nrf2 might directly suppress transcription of NLRP3 inflammasome associated genes, including those coding for NLRP3, proIL-1β, and proIL-1α [19,63]. p62 and PGAM5 are regulators of the Nrf2/Keap1 as well as of the NLRP3 inflammasome pathway. p62 supports activation of Nrf2 [20,23] and antagonizes NLRP3 inflammasome activation [84,85], whereas PGAM5 is required for inflammasome activation [88] and negatively influences Nrf2 activity [33]. Whether both proteins play a major role in the antagonistic crosstalk of Nrf2 and the NLRP3 inflammasome remains to be determined. The reasons why Nrf2 expression is required for full activation of the NLRP3 inflammasome are not understood at all, but it might be that both complexes physically interact [63,64]. The antagonistic relationship of Nrf2 and the NLRP3 inflammasome is also reflected by the fast and Keap1-independent degradation of Nrf2 upon NLRP3 inflammasome activation (Figure 6) [63].

The Nrf2 and NLRP3 inflammasome pathways are interconnected at different levels and mainly in an opposite or mutually antagonistic manner, suggesting that this crosstalk is of physiological importance. Both pathways are critically involved in several common inflammatory and neurodegenerative diseases and, in addition, the Nrf2 pathway is involved in cancer. The elucidation of the molecular mechanisms underlying this crosstalk is not only of scientific interest, but might also contribute to the development of novel and better treatment options for patients suffering from the numerous conditions, which involve one or both of these stress-associated pro-inflammatory pathways.

## Figures and Tables

**Table 1:** Compounds, which activate Nrf2 and inhibit the NLRP3 inflammasome in different disease models. It is not established for all substances, whether they activate Nrf2 by the modification of cysteine residues of Keap1 (canonical Nrf2 activation). In part, an inhibition of NF-κB has been observed in these studies. ND: no data.

Compounds	Disease Model	Nrf2 Activation	NLRP3 Inflammasome Inhibition	NF-κB Suppression	Literature
epigallocatechin-3-gallate (polyphenol in green tea)	lupus nephritis	yes	yes	yes	[90]
antroquinonol from fungal *Antrodia camphorata* (folk medicine in Taiwan)	IgA nephropathy	yes	yes	ND	[91]
osthole (from *Cnidiummonnieri* (L.) Cusson seeds)	IgA nephropathy	yes	yes	yes	[92]
sodium arsenite (NaAsO_2_), arsenic trioxide (As_2_O_3_-Trisenox)	peritonitis	not shown in the study, but well established	yes (plus NLRP1 and NLRC4)	ND	[93]
mangiferin (flavonoid)	sepsis-induced acute kidney injury	yes	yes		[94]
SMA-12b (sulfone, similar to phosphorylcholine from glycoprotein of *Acanthocheilonema viteae*)	collagen-induced arthritis	yes	yes	yes	[95]
prostaglandin 15d-PGJ_2_	peritonitis	not shown in the study, but well established	yes (plus NLRP1)	ND	[66]
sulforaphane (from vegetables such as broccoli sprouts)	peritonitis	not shown in the study, but well established.	yes (plus NLRP1; NLRC4 and AIM2)	ND	[67]
(-)schisandrin B (phytochemical)	peritonitis	yes	yes	yes	[96]
mangiferin (glucosylxanthone from *Mangifera indica*)	acute liver injury	yes	yes	ND	[97]
sulforaphane (from vegetables such as broccoli sprouts)	Alzheimer’s disease	yes	yes	ND	[73]
epigallocatechin-3-gallate (from green tea)	contrast-induced renal injury	yes	yes	ND	[98]
wogonoside (flavonoide from *Scutellaria baicalensis Georgi*)	acute liver injury	yes	yes	ND	[99]
asiatic acid (triterpenoid from *Centella asiatica*)	spinal cord injury	yes	yes	ND	[100]
biochanin A (isoflavone from red clover, cabbage or alfalfa)	acute liver injury	yes	yes	yes	[80]
asiatic acid (triterpenoid from *Centella asiatica*)	spinal cord injury-induced acute lung injury	yes	yes	ND	[101]
minocycline (tetracycline derivative)	diabetic nephropathy	yes	yes	ND	[102]
*Ecklonia cava* polyphenol extract	renal inflammation	yes	yes	yes	[103]
*tert*-butylhydrochinone	ischemia/reperfusion	yes	yes	ND	[79]
luteolin (flavonoid)	ischemia-reperfusion injury	yes	yes	ND	[104]
«compound 1»	DSS-induced colitis	yes	yes (priming)	ND	[74]
citral (from *Litsea cubeba*)	lupus nephritis	yes	yes	ND	[77]
sulforaphane	acute pancreatitis	yes	yes	yes	[105]
dimethyl fumarate	gout	yes	yes	ND	[63]
Berberine (alkaloid)	gouty arthritis	yes	yes	ND	[106]
daphnetin (coumarine derivative)	«mitochondrial dysfunction»	yes	yes	ND	[107]
xanthohumol (flavonoid from *Humulus lupulus L.*)	acute lung injury	yes	yes	yes	[68]
diosmetin (3′,5,7-trihydroxy-4′-methoxyflavone)	acute lung injury	yes	yes	yes	[78]
puerarin (from Kudzu root)	age-related macular degeneration	yes	yes	ND	[108]
isoliquiritigenin (from *Glycyrrhiza uralensis*)	brain injury after intracerebral hemorrhage	yes	yes	yes	[71]
curcumin (from *Curcuma longa*)	osteoarthritis	not shown in the study, but well established	yes	ND	[76]
Zn^2+^	«NLRP3 inflammasome activation in peritineal mesothelial cells»	yes	yes	ND	[109]
mangiferin (from different plants)	early brain injury after subarachnoid hemorrhage	yes	yes	yes	[110]
*tert*-butylhydrochinone	cerebral ischemia reperfusion injury	yes	yes	ND	[72]
carnosic acid (from rosemary)	DSS-induced colitis	yes	yes	yes	[111]
